# Letter to Editor

**DOI:** 10.1007/s11019-019-09913-7

**Published:** 2019-07-15

**Authors:** Lars Ehlers

**Affiliations:** Danish Center for Healthcare Improvements, Department of Business and Management, Aalborg University, Fibigerstraede 11, 9220 Aalborg, Denmark

To the editor,

Ten years ago, I was accused of scientific misconduct regarding a paper (Ehlers et al. 2009) that I wrote with five colleagues for the British Medical Journal, which showed that a national screening programme for men aged 65 years for abdominal aortic aneurysm (AAA) in Denmark was not cost effective. In the following years, I was heavily criticised by certain Danish media outlets. One newspaper headline that I particularly remember was as follows: ‘Lars Ehlers is responsible for more than 2000 deaths each year’. A number of peer-reviewed papers also tried to discredit Ehlers et al. (2009) paper, and one of those papers (Ploug et al. 2014) was published in your journal.

Even though many years have passed, I still think that it is relevant for the readers of this journal to highlight errors and misunderstandings in earlier papers. In this letter, I hope to explain why so many years have passed before I responded to the aforementioned criticisms. Firstly, it is important to note that, as part of a public health technology assessment (HTA) organisation in 2009, I followed instructions from my managers not to engage in the public debate about AAA screening in Denmark. Now, 10 years later, I am no longer employed by that HTA organisation; thus, I am free to speak out.

The points of contention include the following: Ploug et al. (2014) argued that Ehlers et al. (2009) paper was not a ‘neutral’ scientific analysis but was rather ‘a deliberate attempt to influence decision makers in ways that are similar to popular “nudging” techniques’. They also argued that the paper ‘played a key role in the political decision process’ not to implement AAA screening in Denmark.

Both of these suggestions are incorrect. The paper was only a biproduct of an official Danish HTA report that had been published 1 year prior. That HTA report from 2008 played a key role in the decision process (as it should), while Ehlers et al. (2009) paper did not. Instead, it was a scientific contribution.

The order of events was as follows: in 2007, structural reform in Denmark closed down 13 Danish counties and formed five new organisational entities (called regions), which were responsible for the public health care system. Some years before this, a decision had been made in the former Viborg County to introduce the AAA screening programme for men. The investments and preparations for this screening programme had already begun when, in 2007, the new healthcare management team decided to put this on hold. They demanded a new HTA report as input for a new decision process about whether to introduce this screening programme in the larger geographical area of the Central Denmark Region.

It was an unfortunate situation for the cardiovascular surgeons in Viborg, whose professional research carrier and personal investments were suddenly jeopardised. Their interest in using political means to ‘nudge’ the decision was understandable. The cardiovascular surgeons from Viborg involved the Danish Society of Cardiovascular Surgery, and this interest organisation led the personal accusations of scientific misconduct and were strongly involved in the public debate in Denmark. For obvious political reasons, they did not accuse the official HTA report but rather Ehlers et al. (2009) paper. In addition, for the same political reasons, they created the story that the economic findings in Ehlers et al. (2009) paper were responsible for the political decision not to introduce AAA screening in Denmark.

In 2007, the task of producing the HTA report was handed to the HTA organisation in the Central Denmark Region. As an employee of this organisation, it was my job to perform an economic evaluation (because an initial review had found no good economic evidence that was relevant for Denmark). HTA organisations exist all around the world to provide unbiased evidence to help decision makers in health care. The HTA organisation in the Central Denmark Region was part of the national network of HTA organisations and a part of the international HTAi community, which has a strong identity as a part of an evidence-based medicine movement for the benefit of citizens and patients. At no point in my career as an HTA advisor did I observe either my managers or any other political party trying to ‘nudge’ results from economic evaluations or other HTA findings in any direction.

Ploug et al. (2014) compared key input values from the economic model in Ehlers et al. (2009) paper with other evidence found in the literature. They used this comparison as proof of deliberate choices that were made with the purpose of influencing policy. In addition, they stated that Ehlers et al. (2009) paper did not ‘include the full range of relevant values found in the literature’, and ‘… no methodological or theoretical justification is offered for the choices’.

For context, one must consider that Denmark lacks a long tradition in health economic evaluation, such as what is seen in, for example, the UK. Physicians and economists in Denmark are generally not educated in the science of health economic evaluation, and there is a widespread misunderstanding about the concepts of cost effectiveness, QALY and modelling, even among high-positioned decision makers in health care. Notably, Ploug et al. (2014) are also not experts in health economic evaluation.

Ehlers et al. (2009) paper followed the international guidelines for good modelling practice and applied recommended model validation methods (Weinstein et al. 2003; Philips et al. 2006). Based on a systematic literature search, and in a dialogue with the HTA group, all input values for the economic model were chosen as a set of assumptions, which—together as a mathematical model—were able to reproduce Danish incidence data for the annual number of ruptures, elective surgeries and different causes of deaths (Bech et al. 2008). The cardiovascular surgeons from Viborg were a part of the process of selecting the relevant input values for the Danish model, but they unfortunately left the HTA group when they saw the model’s results. If input values had instead been chosen from the literature without such a validation process, as suggested by Ploug et al. (2014), then the problem of ruptures and deaths would have been vastly overestimated and the value of a screening programme would have been significantly overrated.

The validation methods in Ehlers et al. (2009) paper were described (Ehlers et al. 2009; Bech et al. 2008), and the model was made publicly available on the Internet. As a part of the HTA process, a review of former economic evaluations of AAA screening was also performed and published in English (Ehlers et al. 2008). Other parts of the model’s validation included statistical testing of relevant DRG tariffs as estimates of the costs of surgical treatment (Christensen et al. 2010; Ehlers 2010) and quality of life values after surgery (Ehlers et al. 2011). Therefore, Ploug et al. (2014) incorrectly argued that ‘… no methodological or theoretical justification is offered for the choice of only three out of six categories (of Danish DRG tariffs)’. All these studies were published in scientific journals as biproducts of the publication of the HTA report, including the paper by Ehlers et al. (2009). Ploug et al. (2014) also argued that social, ethical and organisational issues were ignored in the Danish decision process; however, this is also a misunderstanding. The HTA report was structured to provide answers to the World Health Organization’s criteria for screening programmes (Wilson and Junger 1967), and all issues were analysed via scientific methods. Thus, all issues played a key role in the Danish decision process. Furthermore, a series of presentations and discussions between politicians, administrators and researchers were scheduled as a part of the political decision process regarding AAA screening in the Central Denmark Region. This included representatives from the HTA organisation, the surgeons from Viborg as well as other experts in screening (e.g. from the Nordic Cochrane Centre). Rather than providing proof of nudging in Ehlers et al. (2009) paper, Ploug et al. (2014) showed that they were nudged to falsely believe that there was something wrong with the Danish decision process. Specifically, Ehlers et al. (2009) paper was the scapegoat that they were nudged to criticise in public.

The final sentence in the paper by Ploug et al. (2014) is a recommendation that decision makers should be much more critical in their appropriation of scientific advice. Sadly, since 2009, the HTA in Denmark has suffered tremendous setbacks. To my knowledge, we are currently the only European country without a national HTA programme to produce evidence-based decision support in healthcare. In 2009, a letter that was signed by all Danish professors in health economics was sent to The Danish Committee of Scientific Dishonesty, which supported the HTA’s methods for economic evaluation that were used in Ehlers et al. (2009), and they argued that the trial should be dismissed. The Danish Committee of Scientific Dishonesty disregarded this letter, and the trial ran for 3 years before it was finally dismissed. The Danish Governmental HTA funding programme was officially closed in 2011.
